# Supplementary material for: Abscisic Acid and Chitosan Modulate Polyphenol Metabolism and Berry Qualities in the Domestic White-Colored Cultivar Savvatiano
Source: Plants (Basel). 2022 Jun 22;11(13):1648. doi: 10.3390/plants11131648 (PMC9269509; doi:10.3390/plants11131648)
Supplement: Supplementary file 1 [file plants-11-01648-s001.zip › plants-1781367-supplementary.pdf]

# Abscisic Acid and Chitosan Modulate Polyphenol Metabolism and Berry Qualities in the Domestic White-Colored Cultivar Savvatiano

**Dimitrios Evangelos Miliordos [1,2,3,*,†], Anastasios Alatzas [2,†], Nikolaos Kontoudakis [1,4], Angeliki Kouki [1], Marianne Unlubayir [3], Marin-Pierre Gémin [3], Alexandros Tako [2], Polydefkis Hatzopoulos [2], Arnaud Lanoue [3,*,†] and Yorgos Kotseridis [1]**

[1] Laboratory of Oenology and Alcoholic Beverage Drinks, Department of Food Science and Human Nutrition, Agricultural University of Athens, 75 Iera Odos, 11855 Athens, Greece; nickkont@yahoo.gr (N.K.); akouki27@gmail.com (A.K.); ykotseridis@aua.gr (Y.K.)
[2] Molecular Biology Laboratory, Department of Biotechnology, Agricultural University of Athens, 75 Iera Odos, 11855 Athens, Greece; aalatzas@aua.gr (A.A.); alexandrosgtako@gmail.com (A.T.); phat@aua.gr (P.H.)
[3] EA 2106 «Biomolécules et Biotechnologie Végétales», UFR des Sciences Pharmaceutiques , Université de Tours, 31 Av. Monge, F37200, Tours, marianne.unlubayir@univ-tours.fr (M.U.); marin-pierre.gemin@univ-tours.fr (M.-P.G.)
[4] Department of Agricultural Biotechnology and Oenology, International Hellenic University, 1st km Drama-Mikrochori, 66100 Drama, Greece
* Correspondence: dim.miliordos@gmail.com (D.E.M.); arnaud.lanoue@univ-tours.fr (A.L.)
† These authors contributed equally to the work.

Supplementary Data

Table S1: Date of biostimulant applications during 2019 and 2020 vintages.

| | **Biostimulant Application** | | | |
|---|---|---|---|---|
| | **2019** | | **2020** | |
| | Abscisic Acid | Chitosan | Abscisic Acid | Chitosan |
| 1st application | 23st August | 16th August | 27th August | 21th August |
| 2nd application | 27th August | 23st September | 1th August | 27th August |
| 3rd application | 30th August | 30th August | 4th September | 4th September |
| | **Sampling Dates** | | | |
| 1st Sampling | 30th August | 30th August | 4th September | 4th September |
| 2nd Sampling | 10th September | 10th September | 15th September | 15th September |
| 3rd Sampling (Harvest) | 29th September | 29th September | 24th September | 24th September |

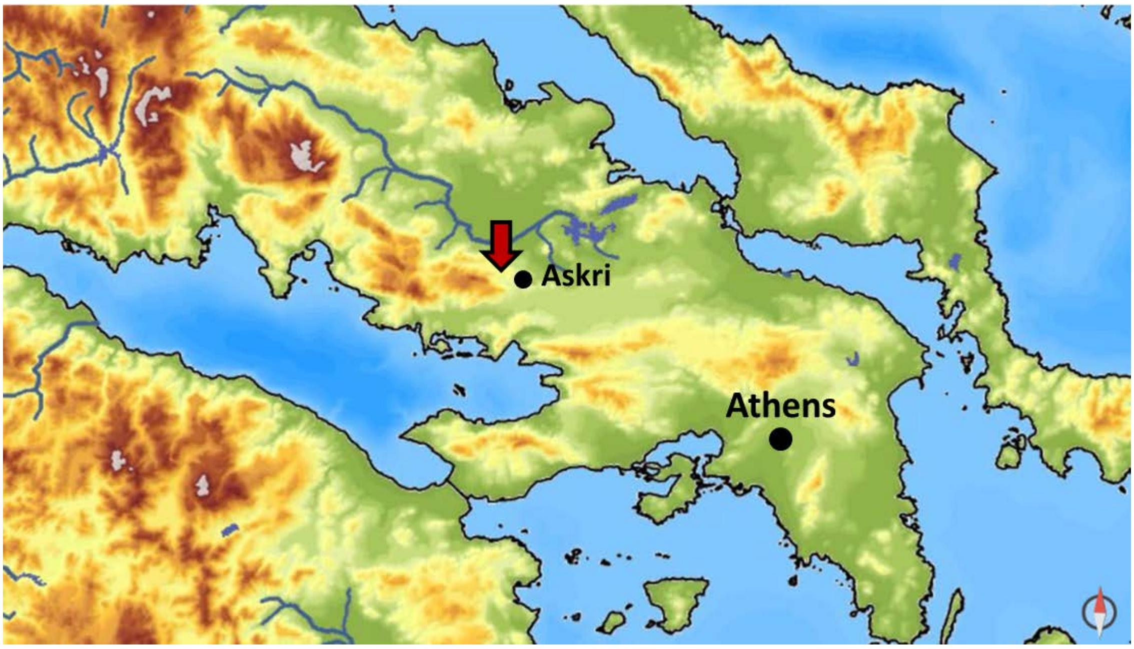


**Figure S1**: Map of Central Greece showing the location of the experimental vineyard

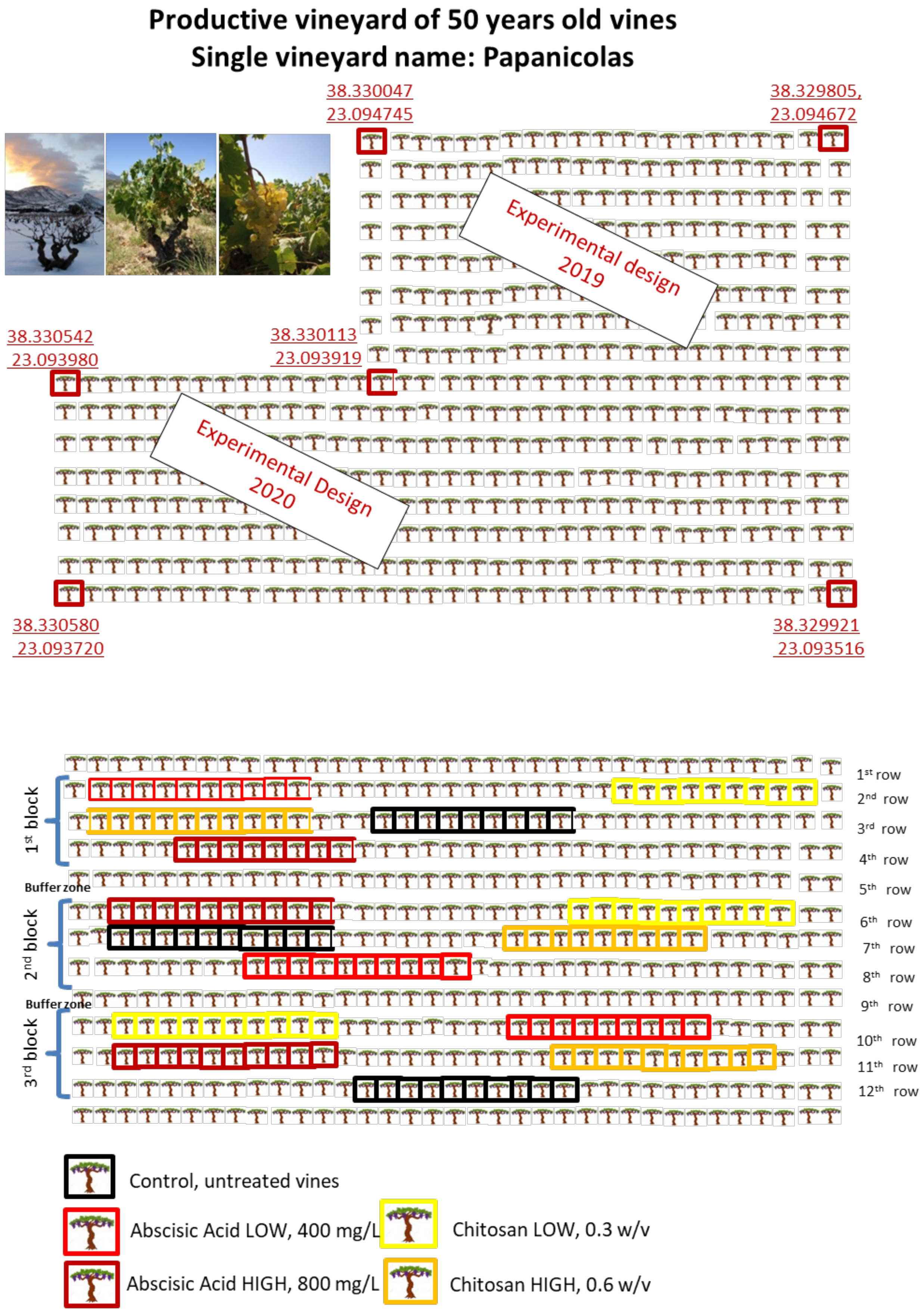


**Figure S2**: Single vineyard in the Muses Valley (A) and the Experimental Design (B)

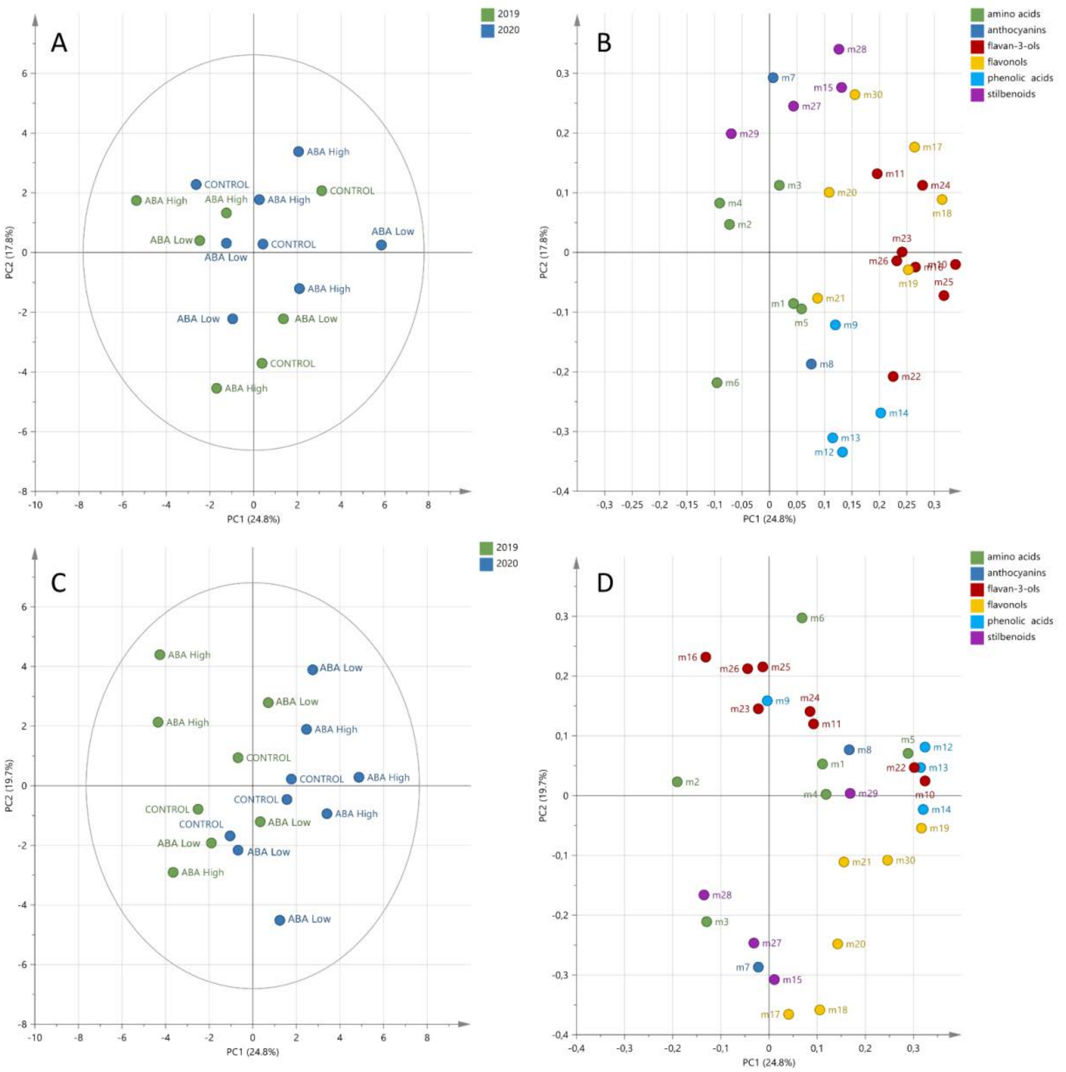


**Figure S3**: Unsupersvised classification using principal component analysis on metabolomic data from grape berries of cultivar Savvatiano at middle veraisson (A, B) and harvest (C, D) stages in 2019 and 2020 treated with ABA. Samples in the score plots (A, C) were colored according to the vintage, and variables in loading plots (B, D) were colored according to the metabolic class. Numbers indicate the ID of metabolites, as follows: L-proline (m1), L-leucine (m2), L-isoleucine (m3), L-phenylalanine (m4), L-tyrosine (m5), L-tryptophan (m6), cyanidin-3-*O*-galactoside (m7), peonidin-3-*O*-(6-*p*-coumaroyl-glucoside) (m8), gallic acid (m9), catechin (m10), epicatechin (m11), coutaric acid (m12), caftaric acid (m13), fertaric acid (m14), E-piceid (m15), catechin-gallate (m16), kaempferol-3-O-glucoside (m17), quercetin-3-*O*-glucoside (m18), quercetin-*O*-glucuronide (m19), quercetin-3-*O*-glucuronide (m20), myricetin-glucoside (m21), procyanidin B1 (m22), procyandin B2 (m23), procyanidin B3 (m24), procyanidin B4 (m25), procyanidin-gallate (m26), *E*-resveratrol (m27), *E*-piceatannol (m28), *E*-ε-viniferin (m29), kaempferol-3-*O*-rutinoside (m30).

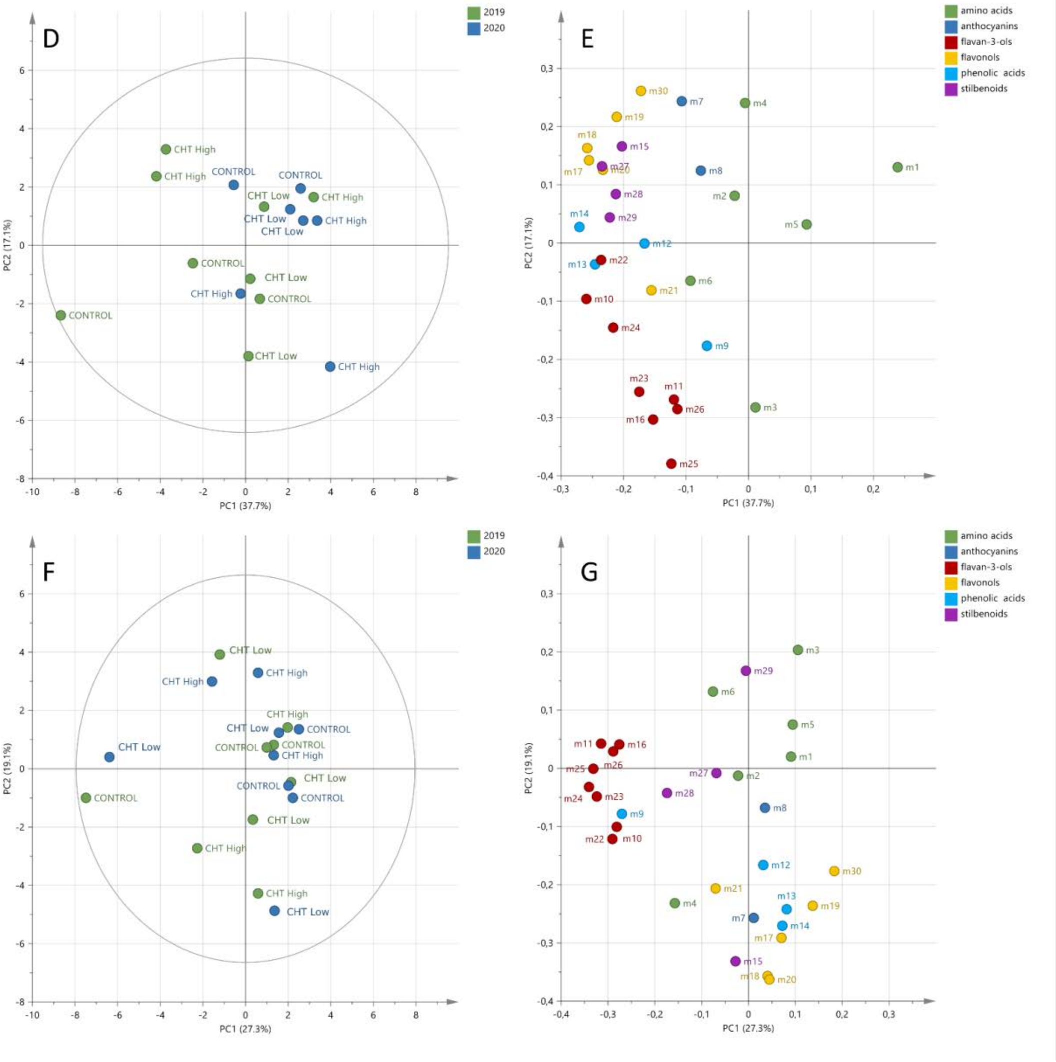


Figure S4: Unsupersvised classification using principal component analysis on metabolomic data from grape berries of cultivar Savvatiano at middle veraison (A, B) and harvest (C, D) stages in 2019 and 2020 treated with chitosan. Samples in the score plots (A, C) were colored according to the vintage, and variables in loading plots (B, D) were colored according to the metabolic class. Numbers indicate the ID of metabolites, as follows: L-proline (m1), L-leucine (m2), L-isoleucine (m3), L-phenylalanine (m4), L-tyrosine (m5), L-tryptophan (m6), cyanidin-3-O-galactoside (m7), peonidin-3-O-(6-p-coumaroyl-glucoside) (m8), gallic acid (m9), catechin (m10), epicatechin (m11), coutaric acid (m12), caftaric acid (m13), fertaric acid (m14), E-piceid (m15), catechin-gallate (m16), kaempferol-3-O-glucoside (m17), quercetin-3-O-glucoside (m18), quercetin-O-glucuronide (m19), quercetin-3-O-glucuronide (m20), myricetin-glucoside (m21), procyanidin B1 (m22), procyandin B2 (m23), procyanidin B3 (m24), procyanidin B4 (m25), procyanidin-gallate (m26), E-resveratrol (m27), E-piceatannol (m28), E-ε-viniferin (m29), kaempferol-3-O-rutinoside (m30)

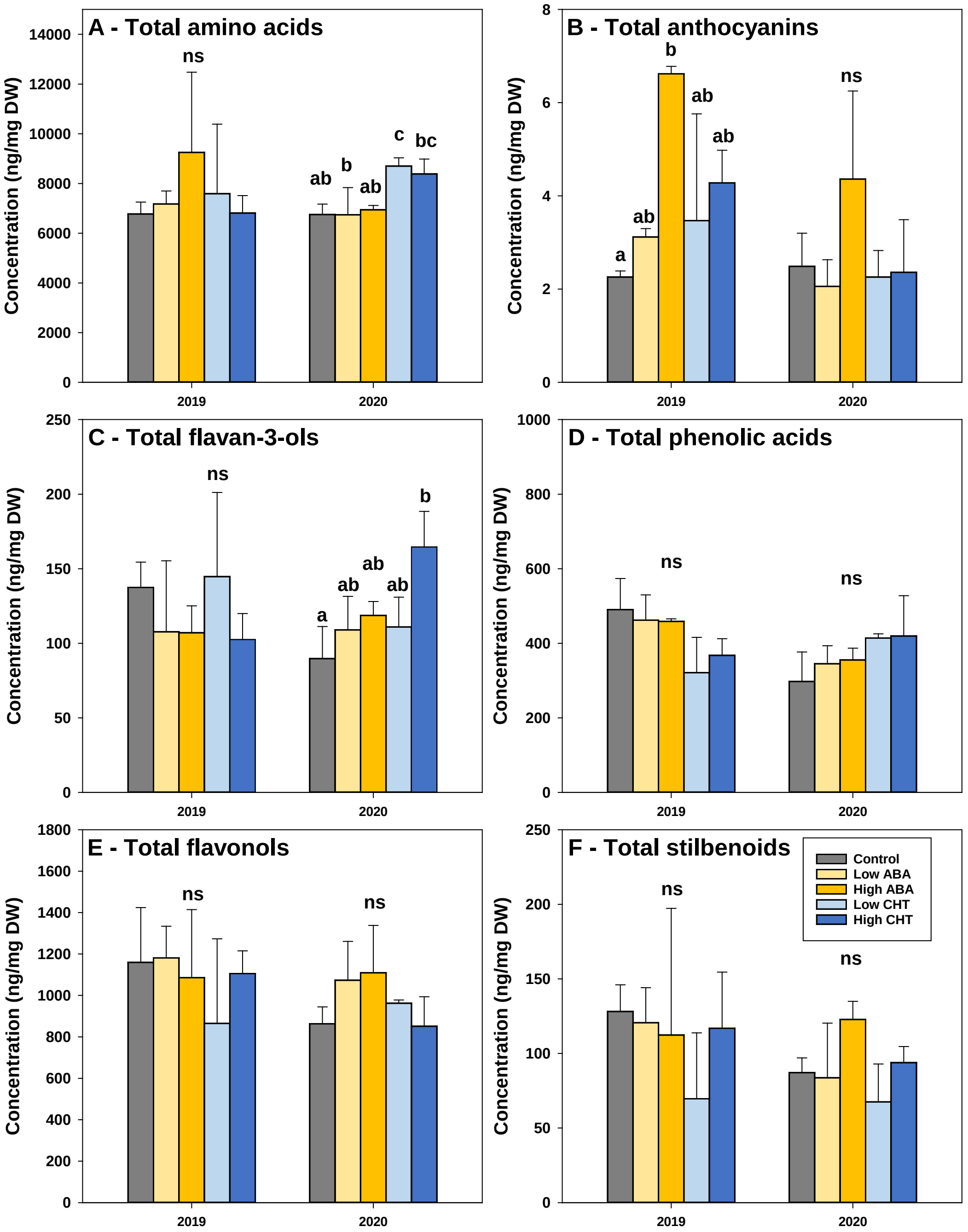


**Figure S5**: Total concentrations of amino acids (A), anthocyanins (B), flavan-3-ols (C), phenolic acids (D), flavonols (E) and stilbenoids (F) in Savvatiano berries at middle veraison stage in 2019 and 2020 depending on the treatment: control (grey), low concentration of abscisic acid (light yellow), high concentration of abscisic acid (dark yellow), low concentration of chitosan (light blue) and high concentration of chitosan (dark blue). Error bars represent the standard deviations. No significant difference (ns) were found between values with the same letters (one-way ANOVA, p-value > 0.05)

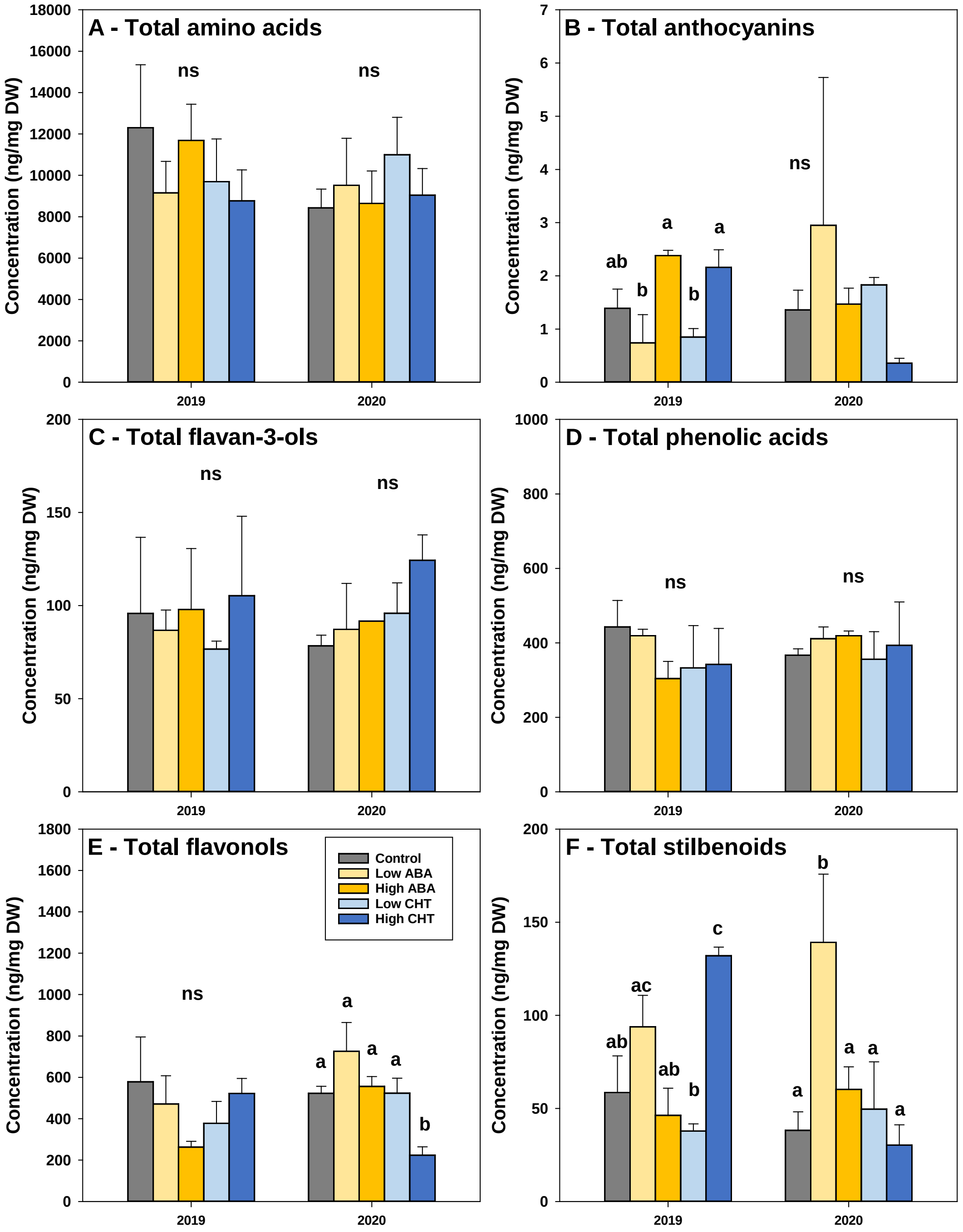


**Figure S6**: Total concentrations of amino acids (A), anthocyanins (B), flavan-3-ols (C), phenolic acids (D), flavonols (E) and stilbenoids (F) in Savvatiano berries at harvest stage in 2019 and 2020 depending on the treatment : control (grey), low concentration of abscisic acid (light yellow), high concentration of abscisic acid (dark yellow), low concentration of chitosan (light blue) and high concentration of chitosan (dark blue). Error bars represent the standard deviations. No significant difference (ns) were found between values with the same letters (one-way ANOVA, p-value > 0.05).

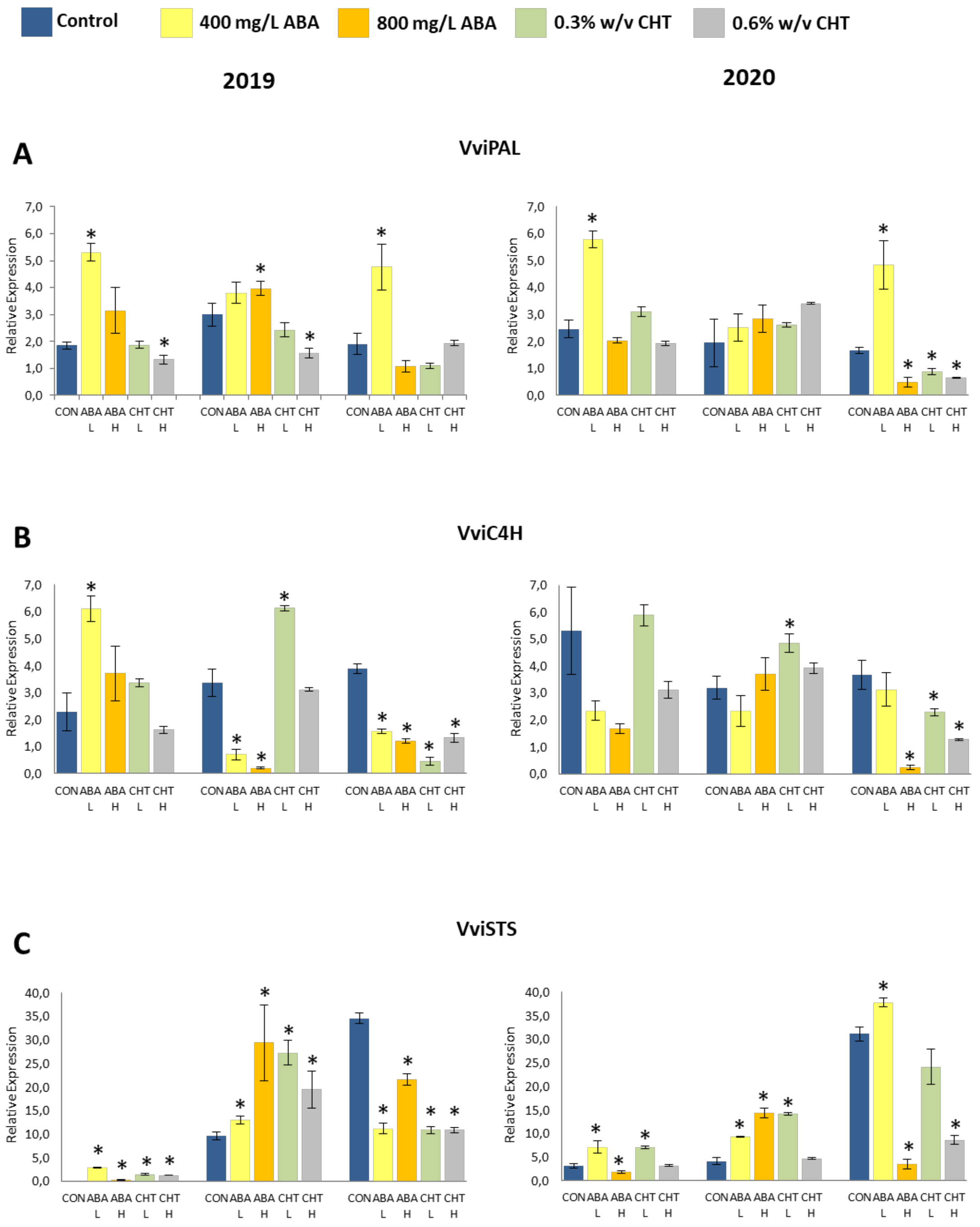


**Figure S7**: Expression level of genes involved in phenylpropanoid pathway (VviPAL (A), VviC4H (B) and VviUFGT (C)) in Savvatiano during two vegetative seasons (2019 and 2020). Vertical bars represent the standard deviation and asterisks indicate the statistically significant differences (Student's *t*-test, p–value < 0.05). The three sampling points (veraison; middle veraison,  and harvest, ) are indicated under each graphs

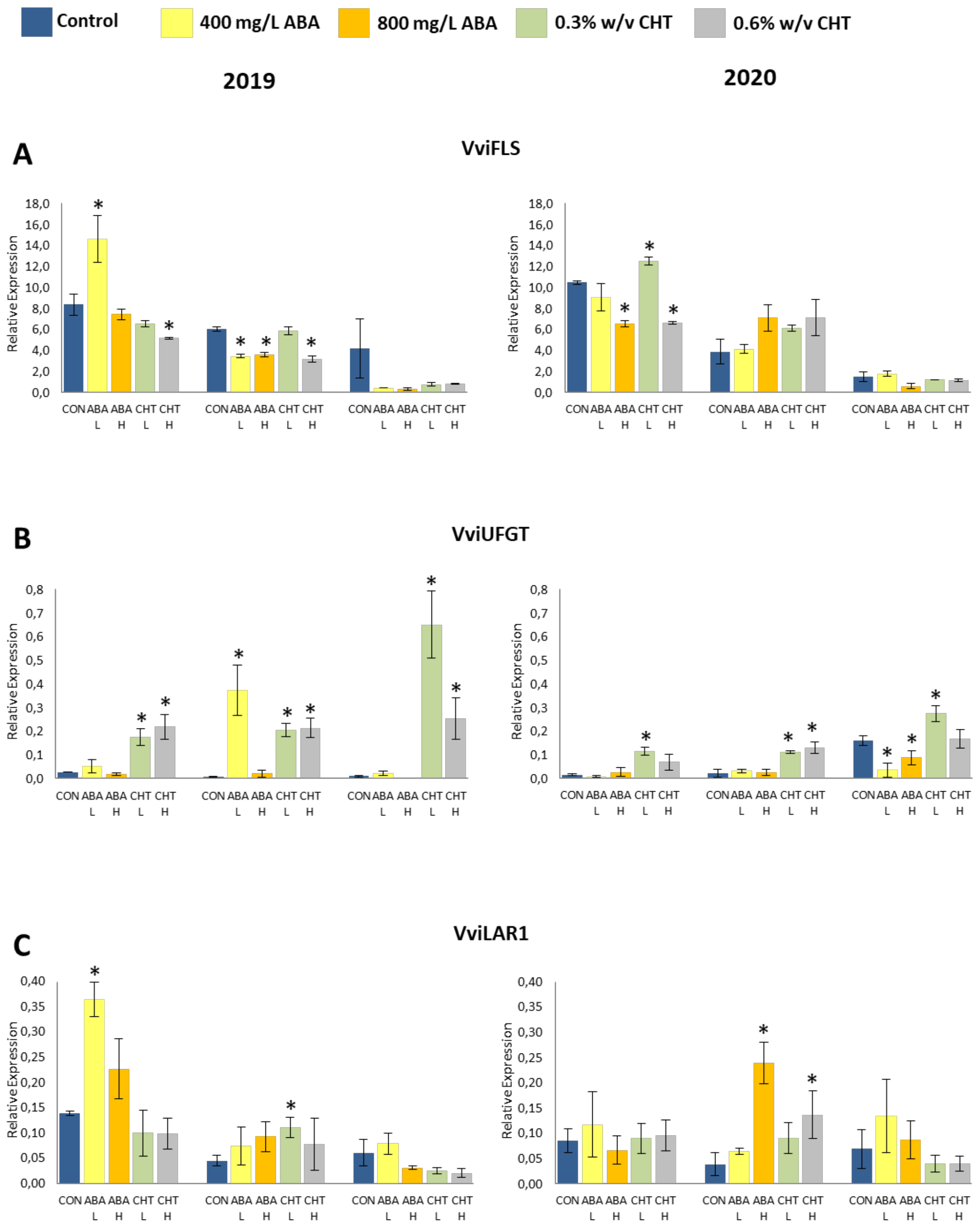


**Figure S8**: Expression levels of genes involved in phenylpropanoid pathway (VviFLS (A), VviUFGT (B) and VviLAR1 (C)) in Savvatiano during two vegetative seasons (2019 and 2020). Vertical bars represent the standard deviation and asterisks indicate the statistically significant differences (Student's *t*-test, p–value $< 0.05$). The three sampling points (veraison; middle veraison, and harvest,) are indicated under each graphs.

Funding: This research has been co-financed by the European Regional Development Fund of the European Union and Greek national funds through the Operational Program Competitiveness, Entrepreneurship and Innovation, under the call RESEARCH–CREATE–INNOVATE (project code: T1EDK-04200 MU-SA).
This work was supported by the Region-Centre Val de Loire (France) grants to CEPATLAS, PRESTO, and INNOCOSM programs as well as by the French Embassy in Athens- Greece with the program "Programme de bourses pour séjour scientifique de haut niveau (SSHN) en France 2020-2021"
